# Harnessing the power of clinical decision support systems: challenges and opportunities

**DOI:** 10.1136/openhrt-2023-002432

**Published:** 2023-11-28

**Authors:** Zhao Chen, Ning Liang, Haili Zhang, Huizhen Li, Yijiu Yang, Xingyu Zong, Yaxin Chen, Yanping Wang, Nannan Shi

**Affiliations:** Institute of Basic Research in Clinical Medicine, China Academy of Chinese Medical Sciences, Beijing, China

**Keywords:** public health, health services, outcome assessment, health care, quality of health care, global health

## Abstract

Clinical decision support systems (CDSSs) are increasingly integrated into healthcare settings to improve patient outcomes, reduce medical errors and enhance clinical efficiency by providing clinicians with evidence-based recommendations at the point of care. However, the adoption and optimisation of these systems remain a challenge. This review aims to provide an overview of the current state of CDSS, discussing their development, implementation, benefits, limitations and future directions. We also explore the potential for enhancing their effectiveness and provide an outlook for future developments in this field. There are several challenges in CDSS implementation, including data privacy concerns, system integration and clinician acceptance. While CDSS have demonstrated significant potential, their adoption and optimisation remain a challenge.

## Introduction

Clinical decision support systems (CDSSs) have evolved significantly over the past few decades,^1 2^ providing clinicians with essential tools for making informed decisions in patient care.^3^ CDSSs have emerged as a promising tool for improving patient outcomes and reducing healthcare costs. These systems utilise electronic health records (EHRs),^4^ medical knowledge databases and advanced algorithms (artificial intelligence (AI), machine learning (ML), etc) to assist clinicians in making more informed decisions by providing evidence-based^5^ and patient-specific recommendations at the point of care.^6–8^ Despite their potential benefits, there are several challenges in CDSS implementation, including data privacy concerns, system integration and clinician acceptance.^9–11^ While CDSS have demonstrated significant potential, their adoption and optimisation remain a challenge.

These systems leverage AI, ML and data analytics to assist clinicians in making more informed decisions by providing evidence-based recommendations at the point of care.^12–15^ While CDSS have demonstrated significant potential, their adoption and optimisation remain a challenge. This review discusses the current state of CDSS, ethical considerations and the opportunities for enhancing their effectiveness, exploring their benefits, limitations, and future prospects.

## Current state of CDSS

### History of CDSS

CDSSs have undergone significant development since their inception, evolving from rule-based expert systems to more advanced AI-driven tools.^16^ This overview traces the history of CDSSs, highlighting key milestones and technological advancements.^17^

**Figure 1:**
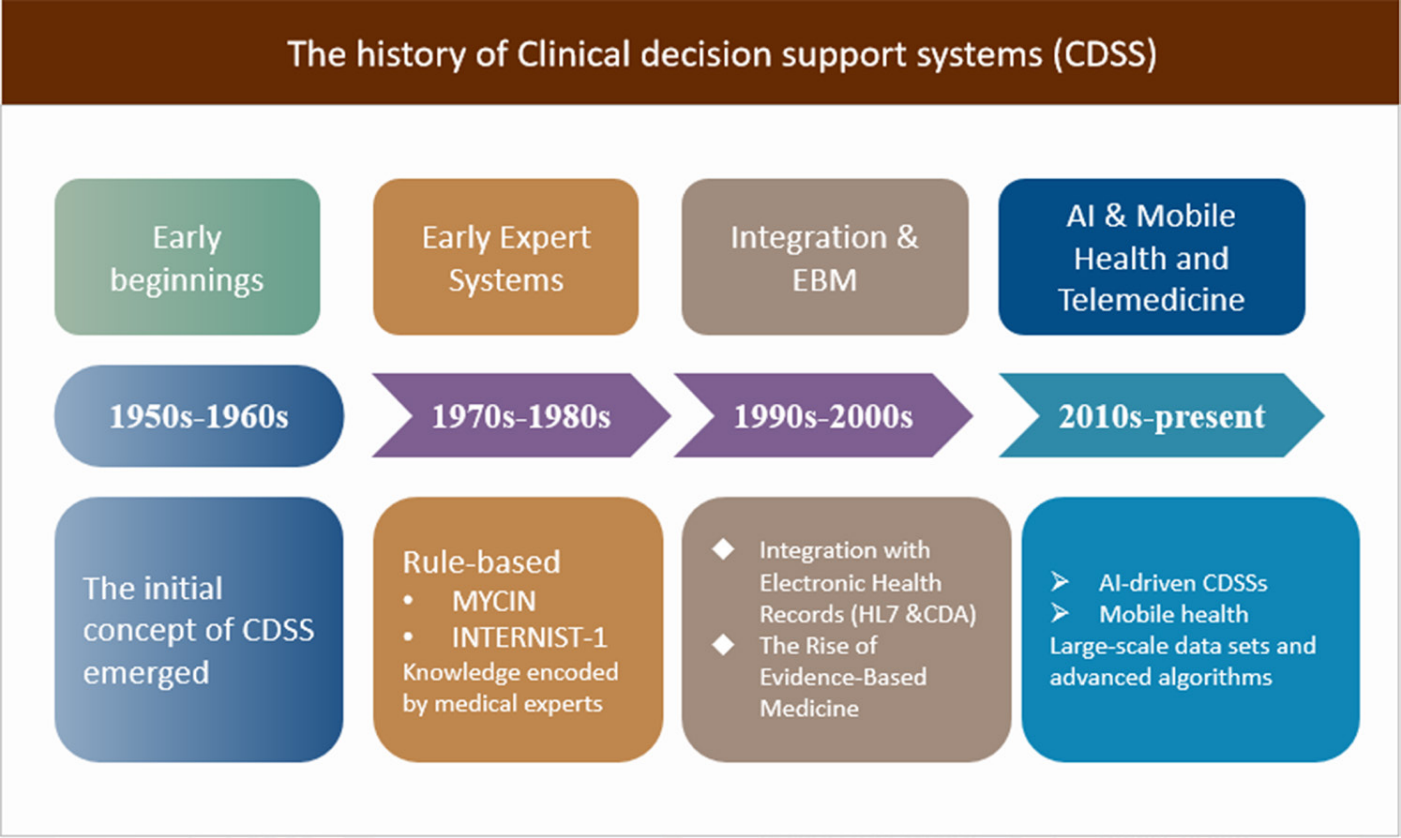
The history of CDSS. AI, artificial intelligence; CDSS, clinical decision support system.

The evolution of CDSSs has been marked by significant milestones and technological advancements, from the early rule-based expert systems to the sophisticated AI-driven tools of today.^18^ As CDSSs continue to evolve, they hold tremendous potential for improving patient outcomes,^19^ reducing healthcare costs and revolutionising the way healthcare providers make clinical decisions^20^ (figure 1)

#### Early beginnings (1950s–1960s)

The initial concept of CDSS emerged with the advent of electronic computers. In the late 1950s, Ledley and Lusted introduced the idea of using computers for medical decision-making in their paper ‘Reasoning Foundations of Medical Diagnosis’. This was a turning point that paved the way for future developments in the field.

#### Early expert systems (1970s–1980s)

The emergence of CDSSs can be traced back to the 1970s, when researchers began developing expert systems using AI techniques. Early examples of these systems include MYCIN, an antibiotic selection support system, and INTERNIST-1, which aimed to assist physicians in diagnosing complex medical cases. These systems were primarily rule-based, relying on knowledge encoded by medical experts in the form of ‘if-then’ rules.

#### Integration with EHRs (1990s–2000s)

As EHRs became more prevalent in the 1990s and 2000s, the integration of CDSSs with EHRs emerged as a priority. This integration enabled more seamless access to patient data, allowing CDSSs to provide context-specific recommendations based on individual patient information. Standards such as Health Level Seven and Clinical Document Architecture were developed during this period to facilitate data exchange between EHRs and CDSSs.

#### The rise of evidence-based medicine (late 1990s–2000s)

The late 1990s saw a growing emphasis on evidence-based medicine, which aimed to use the best available evidence to inform clinical decision-making.^21^ Evidence-based medicine (EBM) is a process of systematically reviewing, appraising, and using clinical research findings to aid the delivery of optimum clinical care to patients. This shift prompted the development of CDSSs that incorporated evidence-based guidelines and clinical practice recommendations, helping clinicians to make decisions based on the latest research findings.

#### Advancements in AI and ML (2010s–present)

The 2010s witnessed rapid advancements in AI and ML techniques, which have significantly impacted the development of CDSSs. By leveraging large-scale data sets and advanced algorithms, these AI-driven CDSSs can provide more personalised and accurate recommendations.^22^ Examples include IBM Watson Health and Google’s DeepMind, which have demonstrated the potential of AI and ML in transforming healthcare decision-making.

#### Mobile health and telemedicine (2010s–present)

With the widespread adoption of mobile technology and the growth of telemedicine, CDSSs have expanded beyond traditional clinical settings.^23^ Mobile health (mHealth) applications and remote monitoring tools have integrated CDSSs to support patients and healthcare providers outside the clinical environment, enabling more proactive and personalised care.

### Development of CDSS

The evolution of CDSS, from its inception to the modern sophisticated systems we witness today, provides a rich tapestry of progress and technological integration. Diving deeper into its development, it becomes evident that the nexus between AI, ML and data analytics plays a pivotal role in this transformation.^24^ With the advent of robust ML algorithms, contemporary CDSSs have transcended these boundaries. These systems now possess the capability to not only process vast datasets^25^ but also refine their recommendations continually, ensuring that they remain relevant and actionable.

As CDSS continue to evolve, research and development efforts should focus on several key areas to maximise their potential impact on healthcare. These areas include:

- Personalised medicine: CDSS can play a significant role in the growing field of personalised medicine,^26^ which seeks to tailor treatments to individual patients based on their unique genetic, environmental and lifestyle factors. Integrating genomic, proteomic and other -omics data into CDSS can help clinicians identify the most effective therapies for each patient, minimising adverse effects and improving treatment outcomes.
- Predictive analytics: The incorporation of predictive analytics into CDSS can enable healthcare providers to anticipate potential complications and disease progression, facilitating early intervention and preventative care. Developing CDSS that can accurately predict outcomes based on historical patient data and other relevant factors will be critical in this regard.
- Natural language processing (NLP): As much of the clinical data stored in electronic health records is unstructured, advancements in NLP can help unlock valuable insights from these sources. By extracting and analysing relevant information from free-text clinical notes, CDSS can provide more comprehensive and accurate recommendations to clinicians.
- Real-time data integration: Integrating real-time patient data from various sources, such as wearable devices and remote monitoring systems, can enable CDSS to provide timely and actionable insights to clinicians. This data can help inform treatment decisions and enhance patient monitoring, ultimately improving patient outcomes.
- Multi-modal data analysis: The analysis of multi-modal data, including medical imaging, laboratory results and patient-reported outcomes, can provide a more holistic view of a patient’s condition. CDSS that can effectively integrate and analyse data from diverse sources will be better equipped to support clinical decision-making.
- Advancements in AI and ML: As AI and ML technologies continue to advance,^27^ CDSS will likely benefit from these developments. The integration of more advanced AI and ML techniques can enable CDSS to process and analyse large volumes of data more efficiently, improve the accuracy of their recommendations and identify previously unrecognised patterns and associations. Future research should focus on developing and evaluating novel AI and ML methodologies for CDSS and exploring their potential applications in various clinical contexts.

In summary, the future of CDSS research and development should focus on addressing current limitations, expanding the use of these systems to diverse settings and adapting to emerging technologies and data sources. By fostering collaboration among stakeholders and exploring innovative solutions, CDSS can continue to evolve and play an increasingly vital role in shaping the future of healthcare delivery.

### Implementation and integration

Implementing and integrating CDSS into existing healthcare systems is a complex process that requires careful planning and execution.^28–31^ Here is a step-by-step guide to help you with the process:

#### Assess the needs and goals

Before selecting a CDSS, it is important to evaluate the specific needs and goals of your healthcare organisation.^32^ Identify the areas where the CDSS can have the greatest impact and determine the desired outcomes.^33^

#### Choose the appropriate CDSS

Evaluate various CDSS solutions available in the market based on their features, compatibility with existing systems, ease of use and scalability. Select a system that aligns with your organisation’s needs, goals and budget.

#### Assemble a multidisciplinary team

Form a team comprising clinical experts, IT professionals and administrative staff to oversee the implementation and integration process. This team should be responsible for developing a comprehensive plan, setting timelines, and ensuring that the project stays on track.

#### Develop a comprehensive plan

Create a detailed project plan, including timelines, milestones and success metrics. This plan should outline the steps needed for successful implementation and integration of the CDSS, such as data migration, system configuration, training, and pilot testing.

#### Data migration and integration

Migrate relevant patient data and integrate the CDSS with existing EHR systems,^34^ ensuring seamless data exchange and interoperability. This step may require collaboration with CDSS vendors and EHR providers to ensure proper integration and data security.^35^

#### System configuration and customization

Configure the CDSS to align with your organisation’s clinical workflows and preferences. Customise the system to accommodate the unique needs of your healthcare setting, such as local practice guidelines, specific diagnostic criteria, and preferred treatments.

#### Training and support

Provide comprehensive training to healthcare professionals who will be using the CDSS. This may include workshops, webinars and hands-on sessions. Develop a support system to address any questions or concerns that arise during the implementation process.

#### Pilot testing

Conduct a pilot test to evaluate the performance of the CDSS in a controlled setting. Use the feedback from the pilot test to refine the system and address any issues before full-scale implementation.

#### Full-Sscale implementation

Roll out the CDSS across the organisation, monitoring its performance and impact on patient care. Continuously evaluate the system’s effectiveness and make necessary adjustments to ensure that it meets the desired goals.

#### Continuous improvement and evaluation

Regularly assess the CDSS’s performance and gather feedback from users to identify areas for improvement. Stay up-to-date with advancements in the field and incorporate new features and updates to ensure that the system remains effective and relevant.

Beyond the aforementioned steps, integrating a CDSS requires a careful understanding of the organisational culture, including the willingness of staff to adapt to change. Recognising that each healthcare setting has its unique set of challenges, whether in terms of infrastructure, patient demographics, or prevailing practices, is pivotal.^36 37^

By following these steps, healthcare organisations can successfully implement and integrate a CDSS into their practice. It is also imperative to understand that integrating CDSS does not negate the significance of human intuition and judgement. In fact, the efficacy of CDSS is maximised when human expertise synergises with technology. Regular feedback loops, wherein clinicians and healthcare professionals provide insights about the system’s functionality, can be instrumental in refining CDSS.^38 39^

The future of CDSS will likely involve further advancements in AI^40^ and ML. By staying attuned to these developments and continuing to address the challenges and opportunities outlined in this article, healthcare organisations can harness the full potential of CDSS to enhance patient care and optimise healthcare delivery.

Moreover, as technology continues its rapid advancement, ensuring the CDSS remains updated is paramount. This includes software updates for improved functionality, incorporating new research findings to keep the decision-making process current and integrating with newer patient care technologies.

### Benefits of CDSS (PRECISE-CARING)

**Figure 2:**
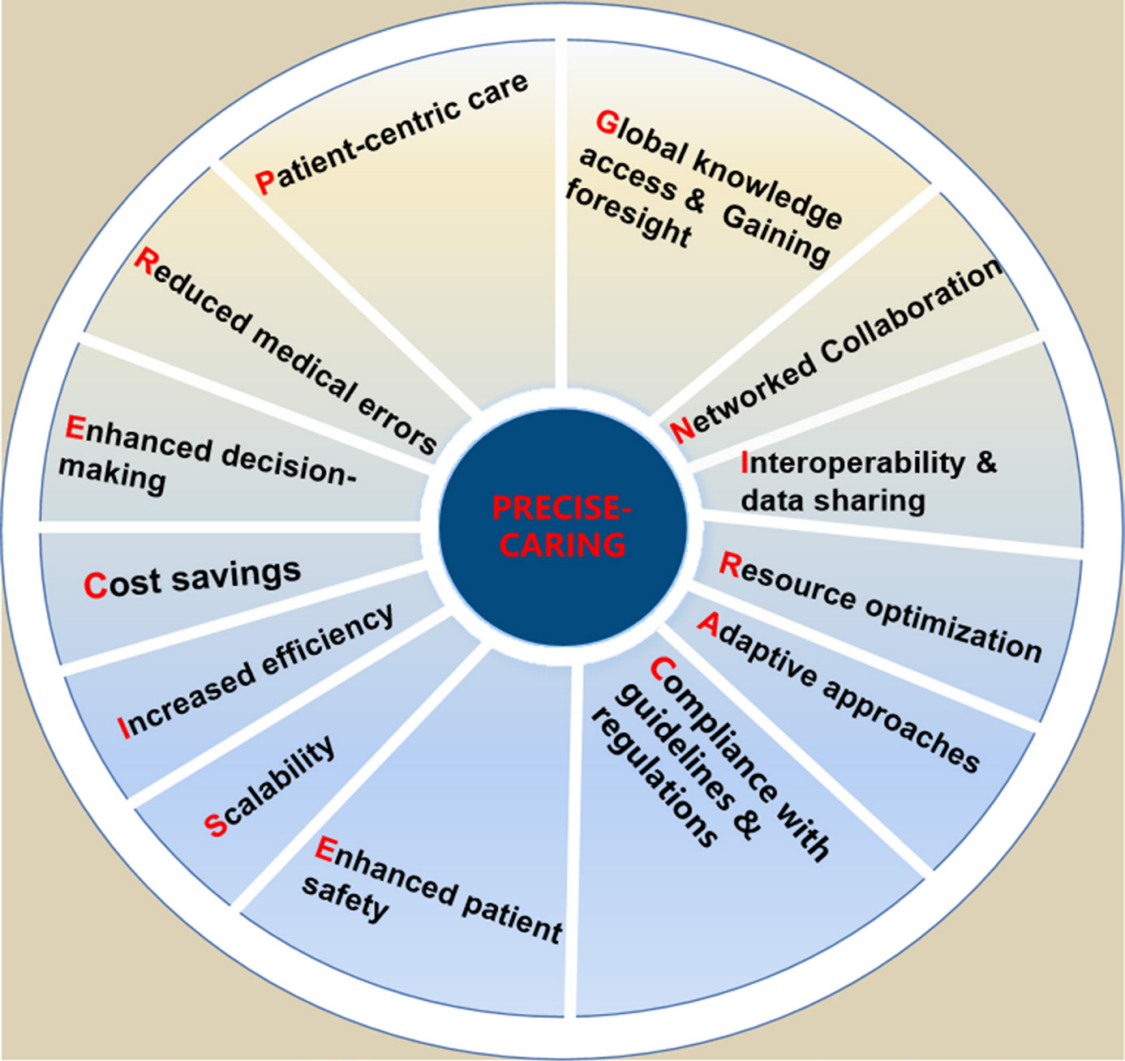
PRECISE-CARING for the benefits of CDSS. CDSS, clinical decision support system.

A CDSS is a health information technology tool that provides doctors, nurses and other healthcare professionals with clinical decision-making support in real-time.^41^ CDSS can assist with diagnosis, treatment and care management by leveraging patient data, evidence-based guidelines and best practices.^42^ CDSS have been shown to improve patient outcomes by streamlining clinical workflows, reducing mortality rates and facilitating evidence-based decision-making. They can also enhance clinician satisfaction by providing real-time feedback and reducing cognitive burden.^43^ Moreover, there are numerous benefits of CDSS,^44^ including patient-centric care,^45 46^ reduced medical errors,^47^ enhanced decision-making,^48–50^ cost savings,^51^ increased efficiency,^5 52–54^ scalability,^55–57^ enhanced patient safety,^58–60^ compliance with guidelines and regulations,^61 62^ adaptive approaches,^5^ resource optimisation,^63 64^ interoperability and data sharing^17 65 66^, networked collaboration,^67–69^ global knowledge access and gaining foresight^70–72^ (PRECISE-CARING) (figure 2).

- Patient-centric care: CDSS facilitates the delivery of personalised care by providing tailored treatment recommendations based on each patient’s unique circumstances and medical history.
- Reduced medical errors: By providing alerts and reminders for potential medication interactions, allergies or contraindications, CDSS can help prevent medical errors and enhance patient safety.
- Enhanced decision-making: CDSS can reduce cognitive overload and human error by assisting healthcare providers in analysing complex patient data, synthesising relevant information and offering tailored treatment suggestions.
- Cost savings: CDSS can help healthcare providers reduce healthcare costs by identifying unnecessary tests, avoiding duplicate procedures and preventing complications that can result from medical errors. By supporting more accurate diagnoses and treatment plans, CDSS can reduce unnecessary tests and procedures, leading to decreased healthcare costs.
- Increased efficiency: CDSS can reduce the time spent on manual tasks, such as searching for information or calculating dosages, by providing quick access to relevant information and automating certain processes. This can save time for healthcare providers and allow them to focus on more critical aspects of patient care.
- Scalability: CDSS can be implemented across various healthcare settings, from large hospitals to small clinics, allowing healthcare providers of all sizes to benefit from improved decision-making and patient care.
- Enhanced patient safety: By reducing the risk of diagnostic errors and adverse drug events, CDSS can help minimise harm to patients and improve overall safety.^73 74^
- Compliance with guidelines and regulations: By incorporating evidence-based guidelines and regulations into the system, CDSS can help healthcare providers stay compliant with industry standards and avoid penalties.
- Adaptive approaches: With CDSS, medical professionals deliver personalised care by customising treatment plans based on individual patient details and medical history.
- Resource optimisation: CDSS streamlines healthcare tasks and automates certain processes, leading to more efficient resource allocation and reduced time spent on manual tasks for healthcare professionals.
- Interoperability and data sharing: CDSS can facilitate communication between different healthcare systems, ensuring that providers have access to the most up-to-date patient information when making clinical decisions.
- Networked collaboration: CDSS fosters better communication and cooperation among healthcare teams by centralising patient data and providing a platform for sharing insights, ultimately improving patient outcomes.
- Global knowledge access and gaining foresight: CDSS serves as a valuable educational resource, connecting medical professionals to the latest research, clinical guidelines and best practices from around the world. CDSS assists in identifying patients who may be at risk for developing certain conditions, allowing for earlier interventions and potentially preventing more severe health issues in the future.^75^

CDSS offer a wide range of benefits that can be captured by the acronym PRECISE-CARING. By leveraging these benefits, CDSS has the potential to revolutionise healthcare practices and significantly improve patient care quality and outcomes.

PRECISE-CARING serves as a useful reminder of how CDSS can help healthcare professionals make more informed decisions, reduce errors, streamline processes and facilitate collaboration. It also emphasises the importance of adapting to changing patient needs, optimising resource allocation and fostering a continuous learning environment to ensure the most up-to-date and evidence-based care possible.

When considering the comprehensive advantages of CDSS, it is also worth noting the empowerment of patients. As healthcare transitions towards a more patient-centric model, CDSS can significantly improve patient engagement by providing them access to easy-to-understand information, allowing them to be active participants in their care journey.^76^

Furthermore, CDSS reduces variations in practice, ensuring that irrespective of the caregiver, patients receive consistent, high-quality care. By flagging potential deviations from best practice guidelines, CDSS ensures a standardised yet personalised approach to care.^77 78^ In addition, as the global health community moves towards value-based care, the role of CDSS in improving healthcare quality while reducing costs becomes more pronounced. It aids in eliminating wasteful spending, optimising resource use and ensuring each patient interaction is maximally beneficial.^79 80^

In summary, PRECISE-CARING highlights the key benefits and clinical significance of CDSS, underscoring the potential for these systems to revolutionise healthcare practices and enhance patient care quality and outcomes.

### Evaluating the impact of CDSS

The current state of evaluating the impact of CDSSs is evolving as technology and methodologies continue to develop.^81^ To determine the true value of CDSS, it is crucial to conduct rigorous evaluations that measure their impact on patient outcomes,^82^ healthcare processes^9 83^ and costs. These evaluations should involve the use of appropriate research designs and methodologies, such as randomised controlled trials, observational studies and cost-effectiveness analyses. The results of these evaluations can be used to inform decision-making and identify areas for improvement in CDSS design and implementation.

As CDSS adoption increases in healthcare settings, evaluating their impact becomes more crucial for ensuring positive outcomes and optimising system performance. The evaluation process is comprehensive, involving multiple factors such as clinical effectiveness, user satisfaction, cost-effectiveness and integration with existing workflows. This requires a combination of quantitative and qualitative methods to assess the CDSS’s impact accurately.

While the existing methodologies for evaluating CDSS are robust, considering the global variances in healthcare delivery is crucial. CDSS implemented in a tertiary care hospital in an urban setting might differ significantly in its impact compared with a primary care setting in a rural environment.^84 85^

The evaluation of CDSS impact is an ongoing process,^86^ with healthcare organisations and researchers continuously monitoring system performance, gathering user feedback and making necessary improvements to ensure the system remains effective and relevant.^87^ While several studies have demonstrated the positive effects of CDSS on patient outcomes and clinical efficiency,^88^ more research is needed to assess their long-term impact.^89^ Future studies should examine the effects of CDSS on healthcare costs, patient satisfaction and the overall quality of care, helping to build a stronger evidence base for their implementation in practice.^90^

Furthermore, the onset of global pandemics, like COVID-19, underscores the importance of agility in CDSS evaluations. Such systems should be nimble enough to incorporate new findings rapidly and ensure healthcare providers are equipped with the most recent and relevant information at all times.^91 92^ Moreover, as patient care becomes increasingly digital, the role of cybersecurity in CDSS cannot be overstated. Evaluating the impact of CDSS should also encompass its resilience against cyber threats, ensuring patient data privacy and system functionality remain uncompromised.^93 94^

In summary, the current state of evaluating the impact of CDSS is characterised by an increasing focus on evidence-based methodologies, data-driven analytics, data privacy, standardisation, collaboration and continuous improvement to ensure that these systems contribute to better patient care and improved healthcare outcomes.^95^

## Ethical considerations

The increasing use of CDSS raises a number of ethical considerations,^96^ including concerns related to algorithmic bias, transparency and accountability. Future research should explore ways to address these ethical challenges, such as developing transparent and explainable algorithms,^97^ incorporating diverse patient populations in the development process and establishing guidelines for the responsible use of CDSS in practice.

As CDSS become more prevalent, ethical considerations must be addressed. Issues such as patient privacy, data security and informed consent need to be carefully considered.^98–100^

In conclusion, as CDSS continue to advance and evolve, addressing the challenges in data privacy, system integration and clinician acceptance will be crucial for realising their full potential in improving patient care and reducing medical errors.

## Discussion

CDSS have demonstrated significant potential to improve healthcare delivery, but their widespread adoption remains limited by several challenges.^101^ Overcoming these obstacles will require innovative solutions and sustained commitment from healthcare providers, developers and policymakers.^102^

In this review, we have highlighted the potential of CDSSs to improve patient outcomes, reduce medical errors and enhance clinical efficiency. The discussion emphasises various aspects of CDSS, including their history, development, implementation, and integration, as well as the benefits and challenges associated with their use.

While CDSS hold promise for improving patient outcomes and reducing healthcare costs, the challenges associated with their implementation cannot be ignored.^103^ To overcome these challenges, a comprehensive and systematic approach is required, addressing not only technical issues but also organisational and human factors.

The potential of CDSS to transform healthcare is significant, but the challenges to their adoption and optimisation are substantial. By addressing these challenges and harnessing the opportunities outlined in this article, it is possible to create more effective CDSS that improve patient outcomes and reduce healthcare costs. Future developments in this field should focus on interoperability, transparent and explainable AI, user-centred design, continuous improvement and collaboration.

### Geographical disparities in CDSS implementation and adherence

An often-underemphasised aspect of CDSS implementation is the geographical disparities that influence its adoption and effectiveness. Our affiliation and insights from various landscapes allow us to delve deeper into these nuances.

### Publication bias

One of the most glaring issues is the publication bias that tends to favour high-income, English-speaking countries. The majority of CDSS literature emerges from these regions, potentially overshadowing significant findings and insights from non-English speaking countries. This bias can skew our understanding of CDSS’s universal applicability and challenges. It is vital for future research to actively seek out and incorporate studies and experiences from a wider range of geographical areas to provide a more balanced global perspective.

### Cultural differences

Cultural nuances play a pivotal role in the reception and reliance on CDSS. For instance, certain cultures might lean heavily on traditional medical practices, viewing CDSS recommendations with scepticism. Conversely, in some settings, there might be an over-reliance on technology, potentially overshadowing the clinician’s expertise. Understanding these cultural subtleties is critical to customise CDSS interfaces and recommendations, ensuring better alignment with regional beliefs and practices.

### Training paradigms

The varied clinician training frameworks across different geographical terrains further compound these challenges. Clinicians trained in regions where protocol adherence is paramount might find it easier to trust and follow CDSS recommendations. In contrast, those from more flexible training backgrounds might exercise more clinical judgement, potentially overlooking CDSS insights. Recognising and addressing these training paradigms can better inform CDSS design and integration strategies.

In summary, while the potential of CDSS in transforming healthcare remains undeniable, it is crucial to acknowledge and address the geographical, cultural and educational nuances that influence its global adoption. As we move forward, a more inclusive approach, taking into account these factors, will be instrumental in realising the full potential of CDSS across diverse healthcare landscapes.

## Challenges and future directions

Future research should focus on addressing the current limitations of CDSS, developing new approaches for system integration and exploring novel ways to enhance clinician acceptance.^104^ Additionally, more studies are needed to evaluate the long-term impact of CDSS on patient outcomes,^105^ healthcare costs and clinician satisfaction. As CDSS continue to evolve, they will likely play an increasingly vital role in shaping the future of healthcare.^106^

Challenges associated with CDSS implementation can be broadly categorised into technical, organisational and human factors.^28^ Technical challenges include data quality and interoperability, algorithm transparency and system integration. Organisational challenges encompass resistance to change, financial constraints and regulatory issues. Human factors involve user acceptance, usability, and training.

### Data privacy concerns

Despite their potential benefits, CDSS face several challenges in practice, including data privacy concerns, system integration issues and clinician acceptance. Addressing these challenges requires ongoing collaboration between stakeholders and investment in CDSS development and evaluation.

Data privacy and security are crucial concerns for healthcare providers and CDSS developers.^94^ Ensuring that patient information remains confidential and secure is essential for building trust among clinicians and patients. Some potential solutions to address these concerns include adopting advanced encryption techniques, implementing strict access controls and adhering to relevant regulatory frameworks such as the Health Insurance Portability and Accountability Act (HIPAA).^107–109^

### Clinician acceptance

Clinician acceptance of CDSS is crucial for their successful implementation and adoption. To enhance acceptance, CDSS should be designed with a focus on usability, relevance and non-intrusiveness. Involving end-users in the development process can help to ensure that CDSS meet the needs and preferences of clinicians, ultimately promoting their acceptance and use in practice.^110^

### Incorporating patient preferences and values

Another important area for future CDSS research is the incorporation of patient preferences and values into the decision-making process. By integrating patient-reported outcomes and preferences, CDSS can support shared decision-making^111^ and enhance patient-centred care.^112^ This will require the development of new methodologies and techniques to elicit and incorporate patient preferences into CDSS algorithms effectively.

### Expanding CDSS applications to underserved populations and settings

One of the future directions for CDSS research should involve expanding their use to underserved populations^113^ and settings, such as rural healthcare facilities and low-resource environments. Developing CDSS solutions that are adaptable to varying resource levels and local contexts can help to address healthcare disparities and ensure that the benefits of these systems are accessible to a broader range of patients.

### System integration

Integrating CDSS into existing clinical workflows and electronic health record systems^114^ can be challenging, particularly in complex healthcare settings. Future research should explore innovative approaches to improve system integration,^31^ such as utilising standardised data formats, adopting service-oriented architectures, and employing user-centred design principles.

The successful adoption of CDSS requires a supportive ecosystem, including strong leadership, a culture of innovation and the availability of resources for training and education. Creating this environment may involve the development of policies and incentives that encourage the use of CDSS and foster collaboration among stakeholders.

### Training and education

To ensure the successful adoption of CDSS in clinical practice, clinicians must be adequately trained and educated on the use of these systems. This includes understanding the capabilities and limitations of CDSS, interpreting their recommendations and integrating these recommendations into their clinical decision-making processes.^3^ Healthcare organisations should invest in training and educational resources for clinicians to promote the effective use of CDSS and ensure that their potential benefits are realised.

### Tailoring CDSS to local contexts

One of the essential aspects of CDSS implementation is ensuring that the system is tailored to the specific needs and requirements of the local healthcare environment.^115^ This includes adapting the system to local clinical guidelines, workflows and preferences. Customisation of CDSS can lead to higher user acceptance and better integration with existing practices.

### CAUCICETCI: multifaceted strategies for CDSS advancement

The current state of research on CDSS is vibrant and multifaceted, with ongoing efforts to address various challenges and seize opportunities to enhance its effectiveness in clinical settings.^116^ To enhance CDSS effectiveness, several opportunities can be explored:

- Customisability: Researchers are examining the potential for customisable CDSS to address unique clinical contexts and user preferences, ultimately enhancing CDSS adoption and utility.
- Addressing ethical and legal concerns (responsibility): As CDSS evolves, researchers are exploring ethical and legal issues, such as data privacy, patient consent, and liability, to ensure responsible use and widespread adoption.
- User-centred design: Designing CDSS that are intuitive and easy to use, with an emphasis on reducing cognitive burden and information overload for clinicians.
- Collaboration: Encouraging multi-disciplinary collaboration between healthcare professionals, software developers and data scientists to create more effective CDSS solutions.
- Integration with EHRs: Researchers are working on improving the integration of CDSS with EHRs, focusing on data exchange standards, interoperability and data security. Efforts are being made to create more efficient data flows between systems and ensure real-time access to patient information.
- Continuous updates and knowledge expansion: Research on knowledge management and maintenance for CDSS is ongoing, focusing on efficient ways to incorporate the latest medical research and best practices.
- Evaluation and feedback mechanisms: Studies are being conducted to assess the impact of CDSS on clinical outcomes, user satisfaction and cost-effectiveness, with the goal of improving CDSS design and performance.
- Transparent and explainable AI: Developing CDSS that provide not only recommendations but also explanations for their reasoning, which can help build trust and improve user acceptance.
- Continuous improvement: Incorporating feedback loops and real-time performance metrics to facilitate ongoing system refinement and adaptation to changing clinical needs.
- Interoperability and standardisation: Ensuring seamless integration with EHRs and other healthcare systems through standardised data formats and application programming interfaces.

**Figure 3:**
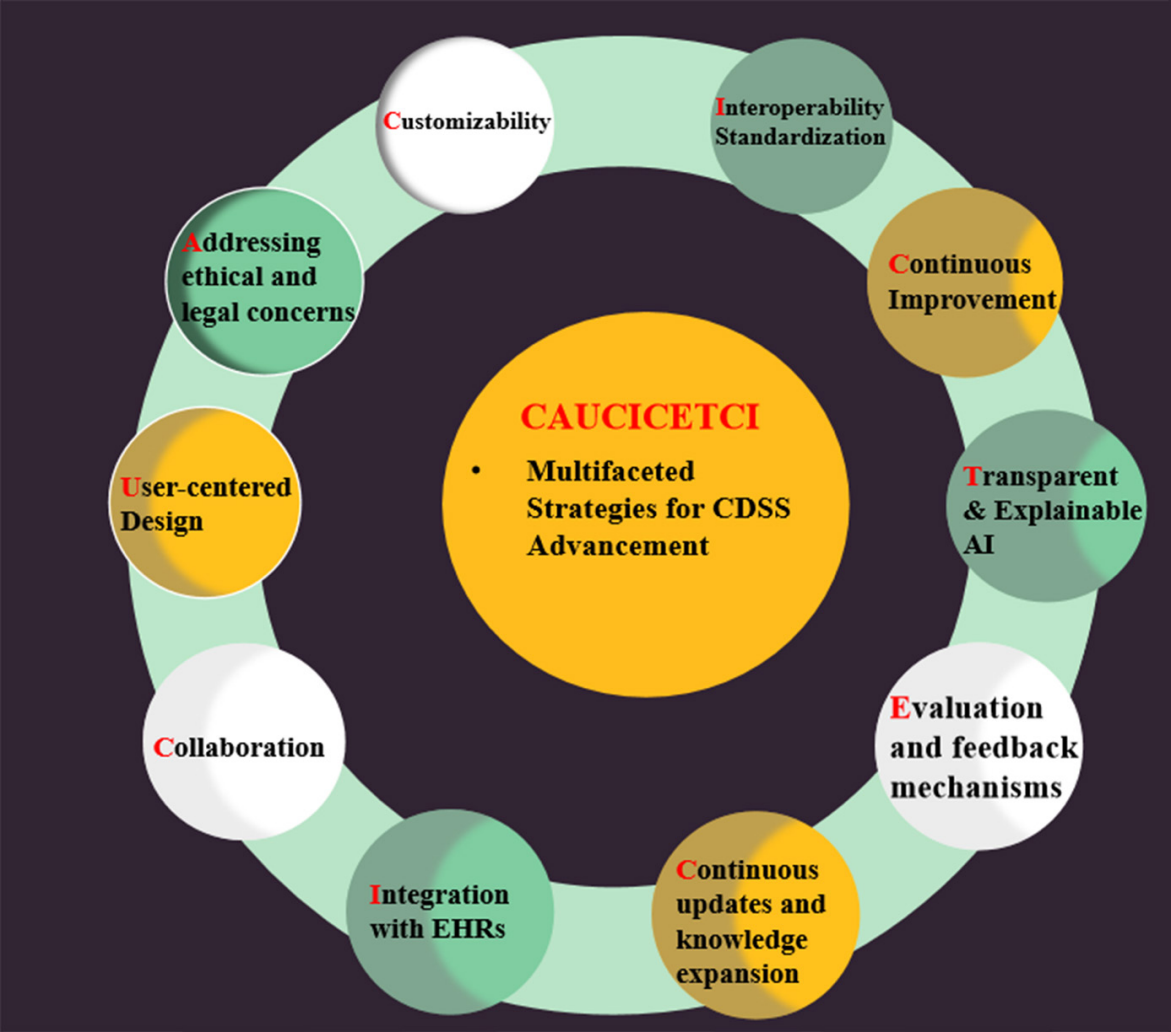
CAUCICETCI: multifaceted strategies for CDSS advancement. AI, artificial intelligence; CDSS, clinical decision support system; EHRs, electronic health records.

By extracting the initials of each phrase and rearranging them, we can form the word ‘CAUCICETCI’ (figure 3), which is related to our topic of improving CDSS effectiveness. In conclusion, the current state of research on CDSS is vibrant and multifaceted, with ongoing efforts to address various challenges and seize opportunities to enhance its effectiveness in clinical settings.

In summary, enhancing the effectiveness of CDSSs relies on addressing key opportunities, such as seamless integration with EHRs, interoperability, leveraging AI and ML, continuous knowledge updates, user-friendly interfaces, customisability, evaluation and feedback, education and training, ethical and legal considerations, and stakeholder engagement.^117^ By focusing on these areas, healthcare providers can ensure that CDSS remains a valuable tool for improving clinical decision-making, optimising patient outcomes, and transforming the overall quality of care.^118^

### Future directions

As technology continues to evolve, the potential for CDSS to advance healthcare will grow. Future developments in AI and ML can further enhance the diagnostic and predictive capabilities of CDSS.^119 120^ Additionally, expanding CDSS applications to underserved populations and settings can help address healthcare disparities and improve access to quality care. Collaborative efforts among healthcare providers, policymakers, researchers and industry partners are crucial to realise the full potential of CDSS in the years to come.

## Conclusion

This review has provided a comprehensive overview of the current state of CDSSs, examining their development, implementation, benefits, limitations and future directions. We have discussed the challenges associated with data privacy, system integration, clinician acceptance, incorporating patient preferences, expanding CDSS applications to underserved populations and the need for training and education. Furthermore, we have explored the opportunities for enhancing CDSS effectiveness through seamless integration with EHRs, interoperability, leveraging AI and ML, continuous knowledge updates, user-friendly interfaces, customisability, and evaluation and feedback mechanisms.

In conclusion, harnessing the power of CDSS requires a multifaceted approach that addresses the barriers to implementation and optimises their effectiveness. By considering the ethical aspects, ensuring seamless integration with other healthcare IT systems, promoting clinician acceptance, focusing on continuous improvement and fostering collaboration among stakeholders, CDSS can become a powerful tool for transforming patient care and improving overall healthcare outcomes.

## Data Availability

No data are available.
